# Oceanic dispersal barriers, adaptation and larval retention: an interdisciplinary assessment of potential factors maintaining a phylogeographic break between sister lineages of an African prawn

**DOI:** 10.1186/1471-2148-8-341

**Published:** 2008-12-24

**Authors:** Peter R Teske, Isabelle Papadopoulos, Brent K Newman, Peter C Dworschak, Christopher D McQuaid, Nigel P Barker

**Affiliations:** 1 Molecular Ecology and Systematics Group, Botany Department, Rhodes University, 6140 Grahamstown, South Africa; 2 Department of Zoology and Entomology, Rhodes University, 6140 Grahamstown, South Africa; 3 Department of Biological Sciences, Macquarie University, Sydney, New South Wales, 2109, Australia; 4 Zoology Department, Nelson Mandela Metropolitan University, P.O. Box 77000, Port Elizabeth 6031, South Africa; 5 Coastal and Marine Pollution, Natural Resources and the Environment, CSIR, P.O. Box 17001, Congella 4013, Durban, South Africa; 6 Dritte Zoologische Abteilung, Naturhistorisches Museum, Burgring 7, A-1010 Vienna, Austria

## Abstract

**Background:**

Genetic breaks separating regional lineages of marine organisms with potentially high broadcasting abilities are generally attributed either to dispersal barriers such as currents or upwelling, or to behavioural strategies promoting self-recruitment. We investigated whether such patterns could potentially also be explained by adaptations to different environmental conditions by studying two morphologically distinguishable genetic lineages of the estuarine mudprawn *Upogebia africana *across a biogeographic disjunction in south-eastern Africa. The study area encompasses a transition between temperate and subtropical biotas, where the warm, southward-flowing Agulhas Current is deflected away from the coast, and its inshore edge is characterised by intermittent upwelling. To determine how this phylogeographic break is maintained, we estimated gene flow among populations in the region, tested for isolation by distance as an indication of larval retention, and reared larvae of the temperate and subtropical lineages at a range of different temperatures.

**Results:**

Of four populations sampled, the two northernmost exclusively included the subtropical lineage, a central population had a mixture of both lineages, and the southernmost estuary had only haplotypes of the temperate lineage. No evidence was found for isolation by distance, and gene flow was bidirectional and of similar magnitude among adjacent populations. In both lineages, the optimum temperature for larval development was at about 23°C, but a clear difference was found at lower temperatures. While larvae of the temperate lineage could complete development at temperatures as low as 12°C, those of the subtropical lineage did not complete development below 17°C.

**Conclusion:**

The results indicate that both southward dispersal of the subtropical lineage inshore of the Agulhas Current, and its establishment in the temperate province, may be limited primarily by low water temperatures. There is no evidence that the larvae of the temperate lineage would survive less well in the subtropical province than in their native habitat, and their exclusion from this region may be due to a combination of upwelling, short larval duration with limited dispersal potential near the coast, plus transport away from the coast of larvae that become entrained in the Agulhas Current. This study shows how methods from different fields of research (genetics, physiology, oceanography and morphology) can be combined to study phylogeographic patterns.

## Background

The marine habitat is considered to contain few physical barriers to dispersal, and marine organisms can therefore potentially disperse over large distances [1,2]. The collection of larvae of coastal marine invertebrates at often great distances from the coast [3], and the often limited genetic structuring of widely separated populations of high-dispersal species [4,5] reinforce this notion. Despite this, sudden breaks in the distributions of marine organisms with potentially high dispersal abilities have been identified throughout the world, and genetic methods have often identified the presence of closely related sister lineages on either side of these breaks [6-8]. Such phylogeographic patterns are often attributed to allopatric divergence resulting from the formation of past land bridges, with subsequent maintenance because propagules of populations on either side of the former barrier primarily recruited to the parent population [6,9]. There are also a number of examples of phylogeographic breaks that cannot be directly linked to geological vicariance events, but for which incomplete isolation of populations in the form of sudden discontinuities in environmental parameters has been invoked. Examples include the freshwater plume of the Amazon-Orinoco outflow that inhibits gene flow of marine organisms between the Caribbean and Brazil [10] and coldwater upwelling in south-western Africa that limits dispersal of tropical and subtropical marine organisms from the Indian Ocean to the Atlantic Ocean [11].

**Figure 1:**
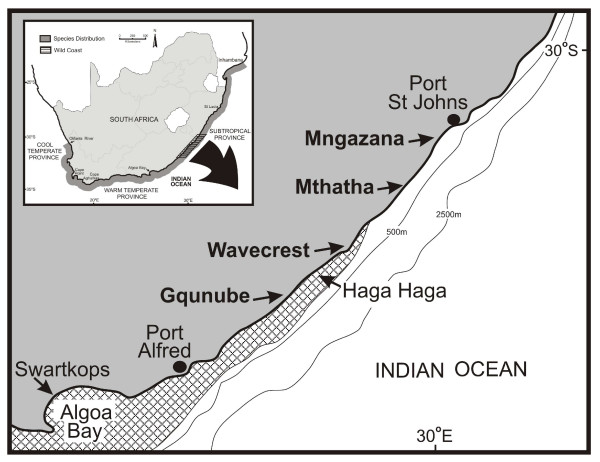
**Map of the sampling area**. Samples of the mudprawn *Upogebia africana *used for genetic analyses came from the four sampling sites indicated in boldface, and those for the larval rearing experiments from the northernmost (Mngazana) and southernmost site (Swartkops). The Haga Haga Estuary is the southernmost site at which mudprawns of the subtropical lineage have so far been recorded, and the Mthatha Estuary the northernmost site at which individuals of the warm-temperate lineage have been found. The chequered area gives the approximate position of cooler water inshore of the Agulhas Current [60]. The insert shows the location of the Wild Coast region in South Africa and the distribution range of *U. africana*.

The South African coastline provides several examples of phylogeographic breaks associated with discontinuities in water temperature. These include breaks at Cape Agulhas and Cape Point, both of which separate lineages associated with cool-temperate and warm-temperate biogeographic regions [12-14], a break in the Wild Coast region on the southeast coast that is associated with the boundary of the warm-temperate and subtropical provinces [13,15-17], and a break in the northeast of the country near St Lucia that separates subtropical and tropical lineages [18,19]. While phylogeographic breaks associated with these biogeographic discontinuities are mostly subtle and are based on significant genetic structure or monophyletic mtDNA phylogroups, the Wild Coast case includes examples of lineages for which morphological [15,16] and physiological (Zardi & Nicastro, in prep.) differences have been identified. The oceanography of the Wild Coast is strongly influenced by the warm, southward-flowing Agulhas Current, which lies about 10 km offshore along most of South Africa's east and southeast coast as it follows the narrow continental shelf [20]. Inshore of the current, there are frequent reversals of current direction, with surface water flow being driven predominantly by longshore wind [21]. From about Port Alfred southwards (Fig. 1), the shelf starts to widen and the current is deflected away from the coast (a region hereafter referred to as the Agulhas Current Deflection Zone, or ACDZ). This results in a decreasing influence of the current that manifests itself in sea surface temperatures being cooler south of this point [22]. A persistent, localised upwelling cell is located inshore of the current where the shelf widens, although it does not always affect surface water temperature [23]. Many tropical and subtropical species have been reported in temperate waters beyond the ACDZ, especially during the summer months, but they do not establish themselves permanently [24].

**Figure 2:**
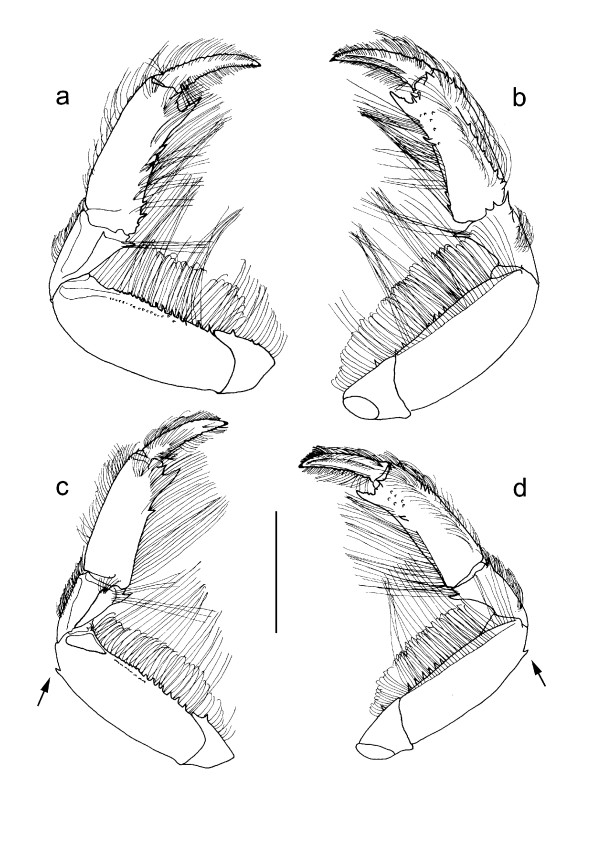
**Right first pereiopods (chelipeds) of *Upogebia africana *(Ortmann, 1894) mitochondrial DNA lineages**. a, b, ovigerous female (cl 21 mm), Keurbooms (NHMW 23876), warm-temperate lineage; c, d, female (cl 19.8 mm), Mpenjati (NHMW 23870), subtropical lineage; a, c, lateral aspect; b, d, mesial aspect. Arrows point to a subdistal spine on the dorsal margin of the merus. The scale bar is 5 mm. Abbreviations: cl, carapace length; NHMW, Naturhistorisches Museum Wien.

The objective of this study was to assess the relative importance of various oceanographic and biological factors in maintaining the separation of genetic lineages of coastal invertebrates at the Wild Coast phylogeographic break. As the study organism, we selected the estuarine mudprawn *Upogebia africana *(Ortmann, 1894), because of all the species that have phylogeographic breaks in this region, its biology has been comparatively well studied. Based on mtDNA sequence data [13], the species can be divided into two major lineages, one associated with temperate biogeographic provinces (with some differentiation between cool-temperate and warm-temperate provinces) and the other subtropical. In previous surveys [13,14], the distribution of the temperate lineage was found to range from the Olifants Estuary in south-western Africa to the Mthatha Estuary on the southeast coast, and that of the subtropical lineage from Haga Haga to Inhambane in Mozambique, with some overlap in the Wild Coast region (Fig. 1). The two lineages can be distinguished on the basis of a single morphological character: the subtropical lineage has a subdistal spine on the first pereiopod that is absent in the temperate lineage (Fig. 2). Dubula and Lasiak [25] further reported that individuals from the central Wild Coast were significantly smaller than those from the southern Wild Coast. The size difference observed may have a genetic basis rather than being merely a function of water temperature, because individuals of the temperate lineage grow larger in warmer water [26].

We investigated how the phylogeographic break between the two mudprawn lineages is likely to be maintained by using a combination of genetic and physiological methods. As temperature is the most obvious environmental parameter that changes along the Wild Coast, we studied the development of larvae of the two lineages at a range of different temperatures. Adult mudprawns are restricted to estuaries and are thus physiologically adapted to tolerate variations and extremes in temperature and salinity, suggesting that changes in sea surface temperatures along the Wild Coast are unlikely to impose strong selection pressure on them. Larval development, however, must obligatorily be completed in the marine environment. We therefore hypothesised that if the lineages are adapted to the temperature regimes characteristic of their biogeographic province, then this would be most readily detectable in the more sensitive larvae.

The influence of the following three factors on maintaining the phylogeographic break was investigated. In each case, a number of expectations in terms of genetic and physiological patterns are listed.

### Dispersal barriers

The core of the southeast African coastal upwelling region is located in the vicinity of Port Alfred, but upwelling may occur as far north as Port St Johns (Fig. 1; [23]). If upwelling represented a significant dispersal barrier, then one would expect the phylogeographic break separating the two mudprawn lineages to be near Port Alfred. The development and survival of larvae should be severely affected at the low water temperatures that may be experienced during upwelling events (≤ 17°C [23]), but would not necessarily differ for the two lineages. In addition, it is possible that the southward-flowing Agulhas Current may limit gene flow from the temperate to the subtropical province. In contrast, larvae of the subtropical lineage can become temporarily entrained in the Agulhas Current may eventually settle in estuaries in the warm-temperate province. Gene flow would then be expected to be mostly unidirectional in a south-westerly direction.

### Local adaptation

Larvae of the two mudprawn lineages may be adapted to temperature regimes characteristic of each biogeographic province, which in nearshore regions range from around 14 to 22°C in the warm-temperate province [27] and from around 20 to >26°C in the subtropical province [28]. A few degrees of difference in mean sea surface temperature between northern and southern Wild Coast may be sufficient to prevent each lineage from establishing itself in the habitat of the other. In terms of genetic structure, one would expect a gradual changeover of lineages along the Wild Coast. In terms of larval development, differences in temperature tolerance ranges and/or optimum conditions for growth and development should be identified.

### Self-recruitment

Recent studies have questioned the notion that the benefits traditionally associated with long-distance dispersal are indeed advantageous in terms of recruitment success [29,30]. Since larvae may disperse away from habitat suitable for settlement, it has been argued that retention near the parent population may be more beneficial since conditions there are obviously suitable for survival (so-called self-recruitment [31]). The larvae of several crustacean species have been shown to employ vertical movement to avoid being swept away from suitable habitats [32,33]. If such mechanisms are employed by mudprawn larvae and contribute significantly towards maintaining genetic structure, one would expect each population to have a unique combination of mtDNA haplotypes. This should manifest itself in a pattern of isolation by distance along the coast.

## Results

### Genetic analyses

Four estuaries spanning the transition zone between the warm-temperate and subtropical biogeographic provinces were selected on the basis of having permanent mudprawn populations at high densities, as established during earlier surveys [13,14]. These were the Gqunube Estuary, a site at the confluence of the Nxaxo and Ngqusi rivers (hereafter referred to as Wavecrest), the Mthatha Estuary and the Mngazana Estuary (Fig. 1). In each of these systems, tissue samples from 35 mudprawns were collected, and a portion of the cytochrome oxidase subunit I gene (COI) was amplified and sequenced.

**Figure 3:**
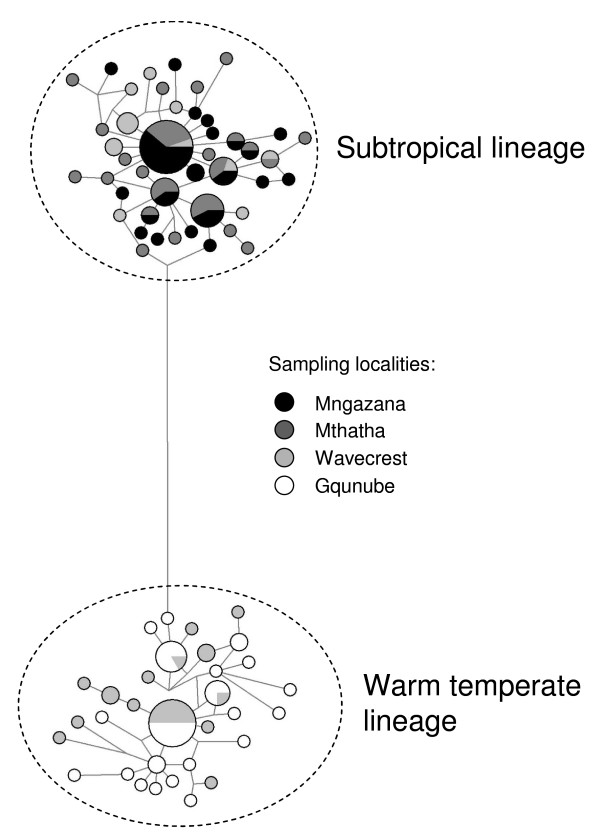
**Network of *Upogebia africana *haplotypes**. A median-joining haplotype network of mitochondrial DNA cytochrome oxidase I (COI) haplotypes of four *U. africana *populations from the Wild Coast, South Africa. The sizes of circles are proportional to haplotype frequencies, the length of lines connecting haplotypes are proportional to nucleotide differences between them, and shading indicates where each haplotype was found.

Haplotypes belonging to the subtropical lineage were exclusively found in the two northernmost populations (Mthatha and Mngazana), and the southernmost population (Gqunube) had only haplotypes from the temperate lineage. The geographically intermediate Wavecrest population comprised a mixture of haplotypes from both genetic lineages (Fig. 3). While haplotype diversity indices were of similar magnitude for all populations (Gqunube 0.94 ± 0.03 [mean ± SD]; Wavecrest: 0.95 ± 0.02; Mthatha: 0.95 ± 0.02; Mngazana: 0.90 ± 0.05), the nucleotide diversity index of the Wavecrest population was considerably higher than those of the others, indicating that the haplotypes present in this population represent a combination of haplotypes from different lineages that are distantly related to each other (Gqunube: 0.009 ± 0.005; Wavecrest: 0.032 ± 0.016; Mthatha: 0.007 ± 0.004; Mngazana: 0.006 ± 0.004).

To test whether gene flow in the Wild Coast mostly takes place in a south-westerly direction (which would indicate that most dispersal of mudprawn larvae takes place while these are temporarily entrained in the Agulhas Current), we used the program LAMARC 2.02 [34] to simultaneously calculate θ (effective female population size multiplied by mutation rate) and M (migration rate). As this program performs best when populations have significantly different genetic structure, we performed exact tests of sample differentiation based on haplotype frequencies. This test identified significant differentiation (P < 0.05) in all pairwise population comparisons, with the exception of the Mthatha vs. Mngazana comparison (P = 0.62). As the Mthatha Estuary is geographically closer to the remaining two estuaries, we excluded the Mngazana population from the LAMARC analyses. Estimates of θ and M for runs using the same three sub-sampled data-sets were consistent, trace plots generated by LAMARC using TRACER[35] indicated that log-likelihood values had reached stationarity following burnin, and the lowest effective sample size found during any run was 388. This indicates that the LAMARC analyses were run for long enough to obtain reliable estimates. Migration rates close to zero were found between the Gqunube and Mthatha populations, in which only temperate and subtropical haplotypes were present, respectively (Table 1). Estimates for the remaining population pairs ranged from 67 for gene flow from Mthatha to Wavecrest to 407 from Wavecrest to Gqunube. Mean estimates of θ were 0.02 for the Gqunube and Mthatha populations, and 0.03 for the Wavecrest population. Given the large standard deviations and confidence intervals, as well as the fact that estimates were based on a single marker, we consider our results sufficient to indicate that gene flow along the Wild Coast is not unidirectional, but insufficient to obtain any more accurate estimates.

**Table 1:** Migration rates among *Upogebia africana *populations

	Gqunube	Wavecrest	Mthatha
Gqunube	-	241 ± 148 *(38 – 698)*	1 ± 0 *(0 – 46)*
Wavecrest	407 ± 390 *(55 – 746)*	-	155 ± 115 *(26 – 415)*
Mthatha	0 ± 1 *(0 – 35)*	67 ± 26 *(1 – 291)*	-

Pairwise migration rates among three *Upogebia africana *populations from the Wild Coast, South Africa, estimated using the program LAMARC (source populations are in rows, receiving populations in columns). Values are mean estimates of migration rate (M) ± SD from three different data-sets comprising 20 individuals per population that were randomly sub-sampled from a complete data-set comprising 35 samples per population. Values in brackets are the means of lower and upper 95% confidence intervals.

To determine whether larval retention in the region may have resulted in genetic differentiation among populations that is correlated with geographic distance, we tested for isolation by distance (IBD) using a Mantel test. As tests for IBD are only meaningful when performed on single regional genetic lineages [14] and require a minimum of three populations (see Methods), only samples from the subtropical lineage were included. No significant correlation was found between genetic and geographic distance among the subtropical lineage haplotypes of the Mngazana, Mthatha and Wavecrest populations (r = 0.0007; P = 0.33), indicating that if larval retention occurs in mudprawns, then it occurs too rarely in the study area to result in genetic structure that can be adequately described by a model of isolation by distance.

### Relationships between genetic and morphological data

Samples were not originally collected with the intention of identifying the subdistal spine on the merus of the first pereiopods, so the relevant portion of the pereiopods was absent in 22 specimens of the subtropical lineage and seven specimens of the temperate lineage. Of the remaining 61 individuals belonging to the subtropical lineage and 50 specimens of the temperate lineage, a single temperate lineage individual from the Gqunube population had the spine, one subtropical individual from the Mthatha population did not have it, and it was not well developed in a small juvenile from the Wavecrest population belonging to the subtropical lineage. An examination of samples collected previously [13,14] revealed that none of the mudprawns collected west of the Gqunube Estuary had the spine (*n *= 85), and that it was present in all of the specimens collected north of the Mngazana Estuary (*n *= 8). These results indicate that the subdistal spine is generally a useful diagnostic feature to distinguish the mtDNA lineages of *U. africana*, but it is likely that there is some interbreeding along the Wild Coast (i.e. the region where the ranges of the two lineages overlap).

### Larval rearing experiments

Larvae of the two mudprawn lineages were reared at various constant temperatures, and their survival and duration of development were compared. Larvae used in these experiments came from two estuaries that are each located well within one of the two major biogeographic provinces inhabited by *Upogebia africana*, the temperate Swartkops Estuary and the subtropical Mngazana Estuary. This choice of populations ensured that in each case, only representatives from a single lineage were studied. In both lineages, the larval sequence comprised three zoeal stages followed by a metamorphic moult to the Decapodid.

**Figure 4:**
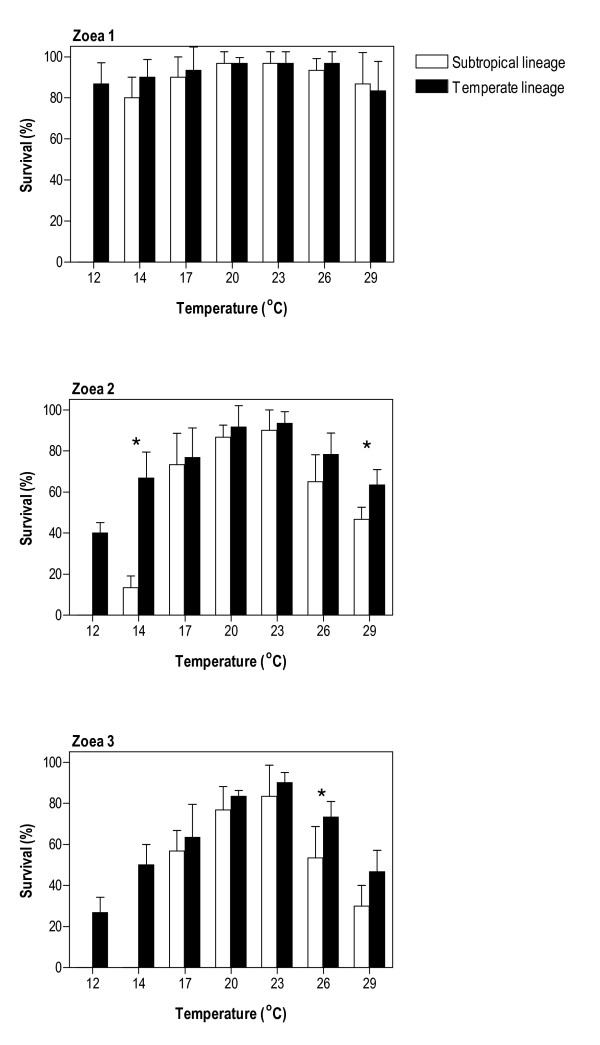
**Survival of *Upogebia africana *larvae**. Cumulative survival (mean + SD) for successive zoeal stages of *U. africana *larvae of the subtropical and temperate lineages as a function of temperature. Asterisks denote a significant difference in survival of larvae from different lineages (based on a Student *t*-test).

*Upogebia africana *larvae of the warm-temperate lineage completed development to the Decapodid at all temperatures (Fig. 4). In contrast, larvae of the subtropical lineage were unable to complete development to the Decapodid at 12 and 14°C (Fig. 4), all Zoea 1 larvae dying at 12°C and those that survived at 14°C subsequently dying during the Zoea 2 stage. Apart from this difference in temperature tolerance, absolute survival at various temperatures was comparable. Larval survival for both lineages was highest at 23°C, decreasing progressively at either side of this temperature. Survival to the Decapodid for the warm-temperate lineage was significantly higher at 26°C, but there were no differences at other temperatures in which larvae from both lineages survived to the Decapodid.

**Figure 5:**
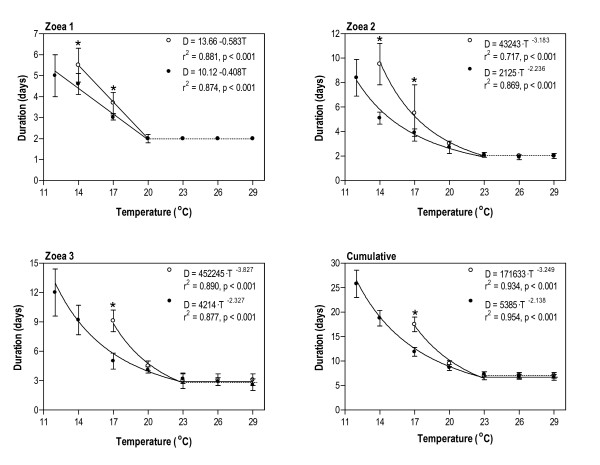
**Larval development of *Upogebia africana *lineages**. Effect of temperature (T) on duration (D, mean ± SD) of successive larval stages and for cumulative larval development of subtropical (white) and warm-temperate (black) *U. africana *lineages. Regressions fitted to temperatures less than or equal to threshold temperature (see text for further details) are given with fitted parameters, coefficients of determination (r^2^) and significance level (p). Asterisks denote a significant difference in duration between lineages (based on a Student *t*-test).

For both lineages the mean duration of development for each zoeal stage and, consequently, also the mean cumulative duration of development decreased sharply with increasing temperature until a threshold temperature was reached, after which duration remained constant or more or less constant (Fig. 5). The threshold temperature for the Zoea 1 was 20°C and for the Zoea 2 and 3 it was 23°C. Each incremental decrease in temperature below these threshold temperatures resulted in a highly significant increase in duration of development. The temperature dependence of duration of development for the Zoea 1 of both lineages at temperatures below the threshold could be described by statistically highly significant linear regressions and for the Zoea 2 and 3 and cumulative duration by power-model regressions (Fig. 5). Slopes of regressions for each zoeal stage and for cumulative duration were significantly steeper for the subtropical lineage (in all cases p < 0.001).

## Discussion

The possibility that regional genetic lineages of a species present on either side of a phylogeographic break may have undergone some adaptive divergence is not usually investigated in studies of this nature, although it has previously been suggested [17,36]. Phylogeographic studies are often exclusively conducted by geneticists, but the most suitable genetic methods available for studying adaptation require complete genome analyses and are only feasible for economically important or charistmatic species [37]. The present study shows that the coupling of methods from different fields of research (genetics, physiology, oceanography and morphology) presents an alternative approach to determine whether genetic patterns identified using presumably neutral markers are linked to environmental discontinuities rather than merely being the result of stochastic processes. The results indicate that the distribution patterns of the two *Upogebia africana *lineages are probably maintained by a combination of environmental and adaptive factors. We therefore suggest that in cases where genetic structure is identified and where environmental differences are evident, the assumption that some local adaptations may exist seems valid and warrants further research into how each lineage is adapted to its environment.

Below, we discuss how each of the three factors hypothesised to be involved in maintaining the Wild Coast phylogeographic break is likely to impact on the two mudprawn lineages.

### Dispersal barriers

Upwelling was identified as a potentially important factor limiting dispersal of mudprawns along the Wild Coast, as cold sea surface temperatures typical during upwelling events negatively affected the development of the larvae of both lineages. The larvae of the subtropical lineage were unable to complete development at the low temperatures experienced during upwelling events, suggesting that few larvae that disperse inshore of the Agulhas Current survive transport towards the warm-temperate biogeographic province during upwelling events. Although the effects of cold water were not as drastic in the case of the warm-temperate lineage, a decreased survival rate was recorded, and larval development took approximately twice as long at 17°C, and even longer at colder temperatures. As prolonged larval duration increases the chances of predation or displacement from suitable habitat, the upwelling region thus represents an incomplete dispersal barrier that primarily influences recruitment success.

The core of the upwelling cell is located south of the southernmost estuary sampled, whereas the point at which the transition zone of mudprawn lineages occurred was identified near Wavecrest. This apparent discrepancy should perhaps not be overemphasized, because upwelling may occur over a large area that stretches from Algoa Bay in the south to Port St Johns in the north (Fig. 1[23]), and changes in the genetic composition of mudprawn populations (haplotypes from the warm-temperate lineage were previously found in the Mthatha Estuary [14]) indicate that the exact position of the transition zone may vary over time. In other coastal invertebrates that are represented in the Wild Coast region by distinct temperate and subtropical mtDNA lineages, the exact positions of phylogeographic breaks or clines differed considerably. In the limpet *Patella granularis*, the ranges of the two lineages overlapped at Coffee Bay (near the Mthatha Estuary [15]), those of the prawn *Palaemon peringueyi *at the Mbhanyana Estuary (south of the Mthatha Estuary [38]), those of the snail *Nassarius kraussianus *at Port Alfred [39], those of the mussel *Perna perna *between approximately Port Alfred and the Haga Haga Estuary (Fig. 1[17]), and a sharp phylogeographic break just south-west of Port Alfred was identified in the cumacean *Iphinoe truncata *[13,14].

Although the genetic data-set used has limitations because a single locus (mtDNA) was sequenced, the fact that bidirectional gene flow was identified indicates that at the relatively small scale at which sampling was conducted, the effect of the southward-flowing Agulhas Current did not seem to impact strongly on gene flow. Oceanographic data indicate that currents in near-shore regions are mostly wind-driven, and that the direction of the wind changes frequently [21,40], suggesting that gene flow among mudprawn populations in this region may mostly occur close to the coast. Genetic data indicate that dispersal by the Agulhas Current becomes important at a larger scale (e.g. over the entire distribution of the subtropical mudprawn lineage), as the migrants that become entrained in the Agulhas Current in the north of the species' distribution and are moved over greater distances have an impact on the genetic diversity of populations downstream [14]. It is also probable that a high proportion of larvae that approach the ACDZ from both south and east and that become entrained in the Agulhas Current are transported away from the coast and are unable reach suitable habitat in time to complete development. This would indicate that the Agulhas Current itself represents a dispersal barrier to both lineages, but the scale of sampling employed in this study is unsuitable to test this hypothesis. Nicastro, Zardi, Roberts and McQuaid (in prep.) released drifters from the eastern south coast during both summer and winter and found that none moved in a north-western direction beyond the Haga Haga Estuary (Fig. 1). All eventually became entrained in the Agulhas Current and were transported away from the coast. Drifters were released ≥ 10 km offshore, and the paths taken may thus not fully indicate how planktonic larvae disperse in the nearshore region. Nonetheless, these results might indicate that larval loss is high in the ACDZ and could further explain why mudprawn larvae of the warm-temperate lineage are unable to establish themselves in the subtropical province.

### Local adaptation

Adaptation of the two mudprawn lineages to environmental conditions prevalent in their biogeographic provinces is likely to constitute an important additional factor limiting their mixing. The lower tolerance of larvae from the subtropical lineage to cold water temperature thus not only results in upwelling presenting a dispersal barrier, but it is also likely to be important in limiting recruitment success of larvae that have reached the warm-temperate province either during periods when there is no inshore upwelling (about 55% of the time at Port Alfred and about 95% of the time at Port St Johns [23]) or that have bypassed the upwelling region while temporarily entrained in the Agulhas Current. Temperatures below 20°C are not uncommon on the south coast even during the summer months [27,28], and the longer duration of development for subtropical mudprawn larvae increases the chance of recruitment failure.

Other adaptive features having a genetic basis that were not studied here may also play a role in limiting the establishment of the subtropical lineage in the warm-temperate province. Firstly, it is possible that adult mudprawns are also less able to tolerate low water temperatures, and individuals that have established themselves in the warm-temperate province may not be able to survive for long. This concept is illustrated by fiddler crab (*Uca *sp.) populations that have their southern limit around the middle of the Wild Coast, establishing populations in estuaries in the eastern portion of the warm-temperate biogeographic province. These populations eventually become extinct during the cold winter months (Newman, pers. obs.). Secondly, the embryos of the subtropical mudprawns that have settled in the warm-temperate province may be affected by the cooler, more saline conditions of those estuaries [41].

Barber et al. [6] suggested that antagonistic behaviour among potential cryptic species of mantis shrimps, *Haptosquilla pulchella*, may contribute to maintaining sharp genetic breaks between regional lineages. Mudprawns are comparatively more limited in terms of social interactions because they are rarely encountered outside their burrows. Moreover, a few exceptions to the trend that individuals from the subtropical mtDNA lineage have the subdistal spine on the first pereiopod, while the temperate lineage individuals do not, may indicate that the two lineages are able to interbreed. This should be tested further using nuclear genetic markers, and if hybrids are identified, then this could be followed by experiments on hybrid fitness. Despite the likely absence of antagonistic behaviour among the lineages, it is nonetheless possible that one lineage competitively excludes the other from its habitat, as mudbanks having the ideal consistency for mudprawn settlement are often extremely crowded. For example, a mean density of 218 ± 166 individuals m^-2 ^has been estimated for the warm-temperate Swartkops Estuary [42]. If recruitment success of the one mudprawn lineage in its region of occupancy is considerably greater than that of migrants from the other lineage, then this will limit the frequency of occurrence of sympatric distribution patterns.

### Self-recruitment

Although no evidence was found for larval retention, it must be acknowledged that mtDNA sequence data have limitations. Recent studies of high-dispersal coastal invertebrates using microsatellites, for example, have identified subtle genetic structure or high autocorrelation values at local scales where none were identified with mtDNA [43,44]. Nonetheless, mtDNA data often provide sufficient signal to detect genetic structure, even in planktonic dispersers within biogeographic regions [45,46]. The lack of genetic structure and isolation by distance found among populations of the subtropical mudprawn lineage indicates that it is unlikely that larval retention is important in maintaining genetic breaks when compared to environmental and adaptive factors.

While there is no clear evidence that larvae of *U. africana *have behavioural strategies that allow them to avoid displacement from suitable habitat, a certain amount of larval retention may nonetheless be possible because of the regional oceanography coupled with a fairly short planktonic development phase. This may contribute to the number of migrants successfully crossing the ACDZ being too low to establish viable populations. Both hypotheses are discussed below.

#### Inshore current flow

The finding that gene flow is bidirectional along the Wild Coast suggests that a large proportion of larvae remain inshore of the Agulhas Current, where wind-driven longshore currents are generally weak and frequently change direction. This suggests that even in the absence of significant isolation by distance (because levels of gene flow are high at this relatively small scale), much larval settlement may nonetheless occur in the vicinity of the parent habitat. McQuaid and Phillips [40] found that 90% of mussel larvae that were dispersed by wind-driven currents on the south coast settled within less than 5 km of the parent habitat, which they attributed to frequent wind reversals. Similarly, in a study on dispersal potential of mangrove propagules by ocean currents using plastic drift cards, Steinke & Ward [47] found that twice as many cards released near Wavecrest were washed ashore in the vicinity than cards released from two more northerly sites (Richards Bay and Durban). This suggests that the oceanography of this region is not conducive to long-distance dispersal. Because the optimum temperature for larval development is the same for the two lineages, adaptive differences are unlikely to play a major role in excluding the warm-temperate lineage from the east coast, and oceanography is a more probable explanation.

#### Short development phase

The larval ontogeny of both lineages comprised three zoeal stages prior to the metamorphic moult. For upogebiid prawns, this is a slightly abbreviated mode of development, since many species pass through four zoeal stages prior to metamorphosis [48,49]. The primary implication of abbreviated development is that larvae are able to complete development within a shorter period compared to sibling species, reducing their exposure to predators and other stressors and, in the context of this study, limiting wide-scale dispersal.

## Conclusion

This study illustrates the value of incorporating information on regional adaptation and morphological differentiation as indicators of evolutionary divergence when studying phylogeographic breaks, in addition to the more commonly applied approach of investigating correlations between phylogeographic patterns and oceanographic features. Warm-temperate and subtropical mtDNA lineages of the mudprawn *Upogebia africana *can not only be distinguished morphologically, but the former was found to be able to complete larval development at low temperatures at which the latter can not. This indicates that the structuring effects of environmental discontinuities and currents not only limit gene flow between the two biogeographic regions, but that the establishment of each lineage in the habitat of the other may be limited by physiological adaptations to regional environmental conditions (possibly including additional ones not studied here). While the work presented here indicates incipient speciation of the two lineages, much work still remains to be done to determine whether the lineages are reproductively isolated, whether there is competitive exclusion, and how embryos and adults are affected by environmental differences between the biogeographic provinces.

Multi-locus and multi-species studies may eventually elucidate how phylogeographic breaks between evolutionary lineages of marine invertebrates in south-eastern Africa became established in the first place. Adaptations to environmental conditions can only arise when units of populations of a species are geographically isolated from other such units, but there are no obvious geological features along the shallow continental shelf in this region that could have acted as long-term vicariant boundaries. A rough divergence time estimate based on mtDNA data indicates that the two mudprawn lineages diverged around 3 million years ago [13], i.e. during a time when the present flow path of the Agulhas Current had been in place for over 2 million years [50]. This suggests that evolutionary divergence of regional lineages may have been driven by oceanographic features similar to the ones observed today, and that the lineages were never completely isolated from each other. Support for parapatric speciation scenarios among populations of marine organisms is still comparatively rare, but may be more common than is generally acknowledged [51,52].

## Methods

### Genetic analyses

Tissue samples from 35 mudprawns each were collected in four Wild Coast estuaries by either breaking off a specimen's right first pereipod (large specimens) or preserving complete individuals (small specimens) in preservation medium containing 70% ethanol and 30% TE buffer. DNA extraction, amplification of a portion of the COI gene and sequencing were performed as described previously using crustacean forward primer 5'-TCA ACA AAT CAY AAA GAY ATT GG-3' and decapod reverse primer 5'-AAT TAA AAT RTA WAC TTC TGG-3' [13]. The sequences generated in this study were submitted to GenBank (accession numbers FJ416903–FJ417042). The program ARLEQUIN 3.1 [53] was used to calculate each population's haplotype and nucleotide diversity based on pairwise differences, and an exact test of sample differentiation based on haplotype frequencies [54] was performed for pairs of populations. To visualise which lineages were represented in each of the estuaries sampled, a median-joining haplotype network was reconstructed using NETWORK 4.2.0.1 [55]. We used the program LAMARC 2.02 [34] to estimate migration rates (M) among populations in conjunction with each population's parameter θ (effective female population size multiplied by mutation rate). A Bayesian Metropolis Hastings Markov chain Monte Carlo search strategy was specified that consisted of a single final chain with 60 000 samples, a sampling interval of 1000, and a burnin of 6 000 000 steps. As MODELTEST 3.7 [56] identified the HKY model [57] as being most suitable for the COI data, we specified a transition/transversion ratio of 5.54 as determined in MEGA 4 [58], as well as empirical base frequencies. Coalescent-based programs perform best when sample sizes are low [59], so we limited our analyses to 20 individuals per population. However, in order to incorporate as much information as possible from the original data-sets of 35 individuals per population, we randomly sub-sampled each population three times to create different data-sets, each consisting of a different sample of 20 individuals per population. Values of θ and M, as well as their standard deviations and 95% confidence intervals, were then reported as the mean values of the three runs. Each run was repeated three times to check for consistency of results, and TRACER v. 1.4 was used to assess whether the program had run for sufficiently long, as indicated by effective sample sizes of no less than 200, and trendlines with stable likelihood values. A Mantel test in ARLEQUIN was used to test for isolation by distance (IBD). Most southern African coastal invertebrates with planktonic dispersal phases comprise mtDNA phylogroups whose distributions are limited by marine biogeographic disjunctions [14]. Pooling sequence data from different regional lineages will therefore incorrectly result in the detection of significant correlations between genetic and geographic distance [e.g., [44]], even in species whose phylogroups exhibit panmixia within their respective biogeographic region. As a minimum of three populations are required to test for IBD, we used only haplotypes of the subtropical mudprawn lineage. One matrix comprised genetic distances (Φ_ST _values based on pairwise differences) and the other geographic distances (shortest long-shore distances among estuarine populations). To test for significant correlation between matrices, we specified 10 000 random permutations.

### Larval rearing experiments

Ovigerous female mudprawns with embryos in an advanced state of development (i.e. with well-developed eyespots) were collected from the muddy intertidal of the lower reaches of the Swartkops and Mngazana estuaries (Fig. 1). In the laboratory, females were held in filtered (0.5 μm), UV-irradiated seawater (salinity of 35, hereafter referred to as culture water) at 20°C under an artificial 12:12 h light:dark (06h00 to 18h00) photoperiod until larvae hatched, which always occurred at night. Actively swimming larvae were collected between 06h00–08h00 the morning after hatching using a wide bore pipette and individually transferred to glass rearing vials filled with 15 ml of culture water at 20°C and fed newly hatched (<8 h old) *Artemia *sp. nauplii. Thereafter, larvae were acclimated to rearing temperatures (12, 14, 17, 23, 26 and 29°C) at a rate of 3°C per hour, by transferring rearing vials in trays between water baths set at the appropriate temperature. After acclimation, three broods of 20 individually reared larvae from the warm-temperate lineage, and three broods of 10 larvae from the subtropical lineage, were reared at each temperature (females from the subtropical lineage were generally smaller and carried fewer eggs).

Daily, larvae were removed from the rearing vials, and examined under a dissecting microscope to determine survival and development stage. Larvae were then transferred to new rearing vials filled with culture water at the appropriate temperature, and fed. Daily analysis continued until all larvae had either metamorphosed to the Decapodid stage or died. Larvae were considered dead when opaque and/or no movement of internal or external structures and appendages could be detected under moderate magnification.

Plots of survival and duration of development data were presented as the arithmetic mean ± standard deviation (Figs 4 and 5). Survival and duration of development for larvae from females of each lineage at each temperature treatment were compared using t-tests (after survival proportions were arc-sine transformed). Quantitative relationships between stage-specific and cumulative duration of development and temperature were described by means of linear and non-linear (power function) regressions. Slopes of regressions were tested for significant differences from zero with *t*-tests, after linearisation of power models by log transformation of both independent and dependent variables. Equality of regression slopes and intercepts was analysed using a procedure equivalent to ANCOVA.

## Authors' contributions

PRT conceived of the study and coordinated the research, collected samples, generated and analysed the genetic data, helped with the larval rearing experiments, and drafted the manuscript. IP collected samples, did larval rearing experiments and helped analyse the genetic data. BKN analysed the larval rearing data and helped draft the manuscript. PCD identified the subdistal spine used to distinguish the two *Upogebia africana *lineages and prepared the drawings of the pereiopods. NPB and CDM provided conceptual guidance and logistical support. All authors contributed to the preparation of the manuscript, and read and approved the final version.
